# Intact primate brain tissue identification using a completely fibered coherent Raman spectroscopy system

**DOI:** 10.1117/1.NPh.5.3.035005

**Published:** 2018-08-17

**Authors:** Damon T. DePaoli, Nicolas Lapointe, Younes Messaddeq, Martin Parent, Daniel C. Côté

**Affiliations:** a Université Laval, CERVO Brain Research Center, Neuroscience, Quebec City, Quebec, Canada; b Université Laval, Center for Optics, Photonics and Lasers (COPL), Physics Engineering, Quebec City, Quebec, Canada

**Keywords:** coherent anti-Stokes Raman scattering, Raman spectroscopy, optical fiber, optical guidance, neurosurgery, deep brain stimulation

## Abstract

Coherent Raman fiber probes have not yet found their way into the clinic despite their immense potential for label-free sensing and imaging. This is mainly due to the traditional bulky laser systems required to create the high peak power laser pulses needed for coherent Raman, as well as the complications that arise from the propagation of this type of energy through silica. Specifically, a coherent anti-Stokes Raman scattering (CARS) probe that could select its integration volume at high resolution, away from the tip of the fiber, is particularly interesting in the case of electrode implantation neurosurgeries, wherein it is possible to place optical fibers on-board the chronic electrode and provide optical guidance during its implantation, through the semi-transparent tip. To this clinical end, we have created an all fiber CARS system, consisting of small, rapidly tunable, turn-key fiber-lasers, capable of creating high wavenumber CARS spectra on the order of tens-of-milliseconds. The use of traditional silica fibers is made possible by the use of the laser’s long pulse-widths (25 ps). The probe itself has an outer diameter of 250  μm allowing it to fit within commercially available metal tubes that can replace deep brain stimulation (DBS) stylets. Using this system, we identified brain tissue types in intact nonhuman primates’ brains and showed the ability to delineate white and gray matters with high resolution. Its advantages over spontaneous Raman stem from the orders of magnitude improvement in spatial resolution, its inherent translatability to three-dimensional (3-D) imaging, as well as the theoretical ability to remove parasitic Raman signal from probe encasements, such as a DBS electrode. The system is planned to have clinical implications in neurosurgical guidance as well as diseased tissue detection.

## 1 Introduction

Coherent anti-Stokes Raman scattering (CARS) microscopy is a well-established technique for studying biological systems in a label-free manner, at video-rate speeds.^1^^,^^2^ Although spontaneous and coherent Raman microscopies both look at inelastic scattering from a sample, caused by inherent molecular vibrations, CARS has the advantage of a greater signal generation, intrinsic three-dimensional (3-D) sectioning (due to its nonlinear nature), and a blue-shifted signal, which allows easy removal of background fluorescent signals.^3^ Although CARS microscopy is a useful tool for probing single molecular vibrations present in a sample, the hyperspectral information routinely acquired in spontaneous Raman microscopy provides much more diagnostic information. To this end, much work has gone into further exploiting CARS to perform hyperspectral imaging. Although hyperspectral CARS imaging is slower than acquiring single bond contrast, it remains much faster than spontaneous Raman imaging. There are several strategies to perform hyperspectral CARS, namely using broadband pulses,^4^^,^^5^ spectrally focused broadband pulses,^6^ or by temporally encoding the signal by scanning either the pump or the Stokes laser.^7^ Broadband CARS (BCARS) creates an entire spectra simultaneously but requires the use of a spectrometer to collect and spectrally decode the generated signal, decreasing its acquisition efficiency and speed in turbid media. Spectral focusing of broadband excitation pulses avoids the need for a spectrometer but relies on a mechanical delay line for vibrational tuning. Wavelength-swept systems allow the use of much more sensitive detectors and provide the ability to perform random access probing of any Raman line within the laser’s tunable bandwidth, giving the potential to drastically decrease the acquisition time if the molecular vibrations of interest are known. Our group previously showed the viability of a laser system using a master oscillator power amplifier pump laser synchronized with a rapidly tunable programmable laser (10 kHz wavelength step rate) to perform wavelength-swept CARS (WSCARS) imaging of biological tissues at much higher speeds than historically possible.^7^

Although coherent Raman (CR) such as CARS or stimulated Raman scattering (SRS) are the tools of choice for imaging, spontaneous Raman has dominated the domain of fiber delivered vibrational sensing, especially in the clinic. This is mainly due to the fact that coherent Raman techniques require high peak power pulses that produce nonlinear effects in dispersive media, diminishing signal generation after a fiber optic. Furthermore, traditional laser systems required for CARS generation are large and designed to be placed on an optical table unmoved. Therefore, translating any of the CR techniques to a clinical fiber-based system requires careful considerations with regard to these nonlinear effects, as well as the portability of the traditionally large lasers required for CR.

Many groups have provided insights into the creation of a fiber-based CARS endoscope for imaging. Légaré et al.^8^ provided the first work of its kind by raster-scan imaging of polystyrene beads in agarose using a 4 mm focusing unit after a 1 m singlemode fiber, used for both light delivery and collection. Balu et al.^9^ examined the nonlinear effects that occur in laser delivery using singlemode silica fibers, photonic crystal fibers (PCF), and large-mode area (LMA) PCFs (<1 m fiber length). They found that the FWM in all fibers overpowered the CARS generation in the sample. Using a filter after the delivery fiber, another multimode fiber (1 m) for signal collection, and a scanning microscope, they succeeded in imaging biological tissue. Brustlein et al.^10^ showed efficient pulse delivery and coherent Raman signal collection from organic crystals using a single double-clad PCF in combination with a microscope after the fiber, to create an “endoscope-like” system with minimal FWM. Saar et al.^11^ carried out coherent Raman imaging using 1 m of polarization-maintaining singlemode silica fibers for pulse delivery in combination with their in-house scanning fiber endoscope (SFE) probe. However, the detection scheme in the backward direction was not ideal, using a 10  mm×10  mm photodiode with a hole cut in the middle for probe insertion. Deladurantaye et al.^12^ created a specially designed LMA, polarization maintaining, double-clad, silica fiber with a microfabricated optical filter on the singlemode core of the fiber, to be used in combination with an SFE probe. Using a collimating and focusing objective after the SFE probe, they succeeded in acquiring images of polystyrene beads deposited on a mirror in the epi-direction. This paper provided valuable insight into the downfalls of micro-optical filters and double-clad fibers for single-fiber CARS generation and collection. Recently, Lukic et al.^13^ performed fiber-based CARS imaging on human skin tissue using a 1 m, 10,000 core, coherent imaging fiber. The probe design has the benefit of imaging with no moving parts; however, the acquisition time is long due to the multicore fiber-inducing substantial pulse deterioration.^14^ Furthermore, the endoscope is 8 mm in diameter making it too large for any neurosurgical procedures.^14^ Most recently, Lombardini et al.^15^ presented a multimodal nonlinear endoscope using a Kagomé-lattice double-clad fiber, miniature objective and microsphere lenses. This probe has the best performance to date in terms of resolution, acquisition time, and signal-to-noise ratio (SNR), however, for use in deep brain surgery, the 4.2 mm outer diameter of the probe is still too large and the design is very complicated.

Although these groups have all focused on coherent Raman imaging endoscopes, our goal is to create a smaller spectroscopic system for tissue identification and classification on-board the implantation of the chronic electrode during deep brain stimulation (DBS) neurosurgery for Parkinson’s disease. Briefly, the surgery consists of a stimulating electrode being implanted with its contact terminals strategically placed deep within the brain in precise structures. The main target brain structure in DBS surgery for Parkinson’s disease, the subthalamic nucleus (STN), is a cell rich region, surrounded by a thin (<1  mm) white matter fiber bundle called the zona incerta. The surgical accuracy of the DBS-lead implantation in the dorsal region of the STN is the overall predictor of treatment outcome, and therefore very high precision optical guidance would be required to improve the surgery. Although we focus on STN-targeted DBS, this relation between surgical outcome and surgical accuracy stands for all DBS procedures. Moreover, the need for better electrode placement is a universal desire in the neurosurgical community and electrode placement is a highly researched topic. For instance, when looking at only unsuccessful DBS outcomes, Okun et al. found that of 40 patients with failed DBS treatment, 19 had misplaced leads.^16^ Moreover, the repositioning of the misplaced implanted leads (some only moved on the order of 1 mm) rescue the treatment’s efficacy with regard to the motor symptoms, quality of life, and global outcome scores.^17^ Our planned technique to improve the guidance of this procedure is to replace the removable stylet, which gives the electrode its rigidity during implantation, with another stylet housing fiber optics. This design limits the probe to a total outer diameter of roughly 300  μm along the entire 29 cm electrode length and therefore makes imaging an unlikely solution.

Spontaneous Raman probes have been presented in the past for brain tissue identification; however, these probes were limited by their spatial resolution when trying to discriminate small brain structures.^18^^,^^19^ The need for the development for clinical CARS probes comes mainly from the substantial increase in resolution (over two orders of magnitude) compared with spontaneous Raman due to its nonlinear signal generation. This increase in resolution not only allows for potential 3-D imaging but also for high-resolution spectroscopy. As noted previously, this is imperative for DBS surgery, as high surgical precision better than 1 mm is critical. Furthermore, CARS has a much higher SNR in high molecular-concentration samples, such as lipid-rich myelinated axons in the brain. This advantage is amplified substantially when considering the spatial volume of acquisition of the two techniques. Fiber-based optical sectioning of spontaneous Raman is achievable; however, the signal will be extremely weak, not only due to the weaker signal generation, but also due to the required small collection-fiber core size needed for confocal detection. These facts along with the ability to generate CARS signal at a desired location away from the fiber tip makes CARS a high potential tool in DBS surgical guidance, wherein optical fibers may be placed within implanted electrodes and sense through the semi-transparent plastic tips.

Our system is composed of an optimal arrangement for decreased nonlinear effects and increased clinical translatability. First to reduce background four-wave mixing (FWM) within the fiber, WSCARS was chosen over BCARS for spectroscopy. Using this modality, we decrease the number of wavelengths present at any moment in the fiber, and consequently decrease the interference between them. To reduce pulse-deteriorating effects, such as self-phase modulation, SRS, and linear dispersion within the fiber, we used a laser system with relatively long (25 ps) pulse widths. Although this decreases our overall signal generation at the sample, it allows the use of commercially available, inexpensive silica fibers with clinically relevant fiber lengths (1  m<L<10  m). Lastly, our custom laser system is compact, turn-key, and fiber terminated, dispensing from free-space optics between source and detection and making the entire arrangement completely portable and ready for the clinic.

This report shows the ability of our complete system to perform CARS spectroscopy in different brain tissue types and ultimately use that detection for high-resolution white and gray matter delineation along probe trajectories in a sample of fixed primate cortex. We specifically show two probe-tip designs, one contact-based with no micro-optical components and one functionalized using a GRIN lens, which adds no additional size to the probe and which produces a focal volume away from the fiber tip. The first design exhibits a very simple probe design that is not only interesting as it defies the theoretical need for high NA focusing units to generate the CARS signal, but also for clinical applications wherein a cheap disposable probe may be of interest. The second design is specifically interesting for DBS guidance wherein we aim to generate our CARS signal on the other side of a plastic electrode, a simple but imperative utility that cannot be achieved using spontaneous Raman. We hope that with future advancements of our system, this information can be used to inform the surgeon in real time of the electrode’s position in the brain during DBS neurosurgery based on the optical response along the trajectory.

## 2 Materials and Methods

### 2.1 Laser System

The laser system was a custom and compact master oscillator power amplifier (MOPA) pump laser at 792 nm synchronized with a rapidly tunable programmable laser (PL) from 1020 to 1044 nm (Genia Photonics, now Halifax Biomedical). These wavelength ranges were chosen to interrogate the high-wavenumber region of a Raman spectra (2800 to $3050\;{\mathrm{cm}}^{-1}$) using CARS. The two lasers have the desired pulsewidth of 25 ps and a 40 MHz repetition rate. The electronics and inner workings of the laser are described in our earlier work.^7^ The only differences in this newer system are the operating wavelength ranges, and the new version of the PL has a maximum 2 kHz wavelength step rate, to decrease jitter. This 2 KHz step rate corresponds to a shortest wavelength dwell time of 1 ms. Tuning of the laser could be performed in a random access manner using preset wavelengths or in an ordered manner by choosing the desired spectral resolution. The highest spectral resolution that could be performed was using a step size of 0.2 nm.

### 2.2 Fiber Optic Delivery

**Fig. 1:**
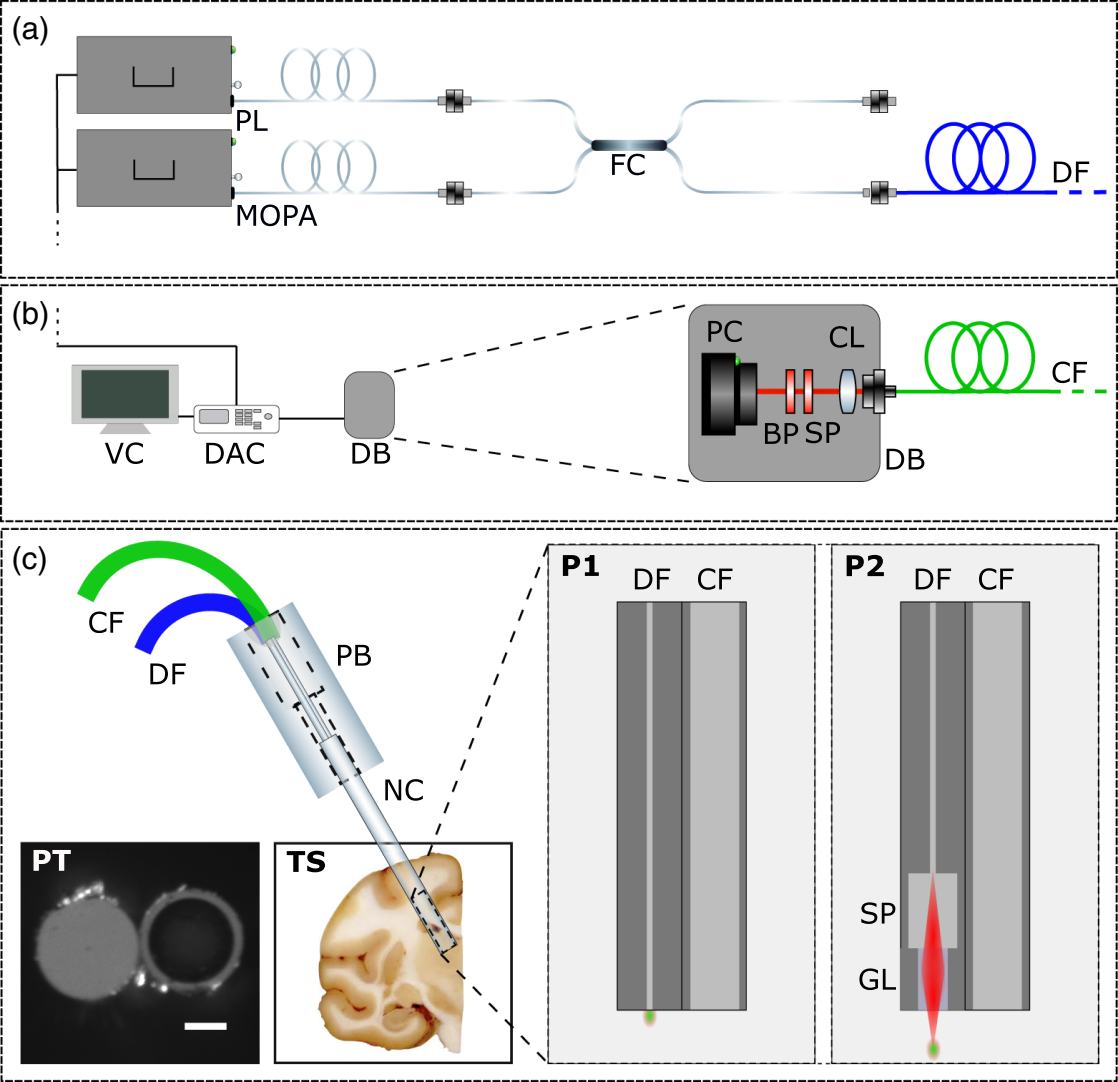
(a) Laser delivery optical layout. Abbreviations: PL, programmable fiber laser; MOPA, master oscillator power amplifier fiber laser; FC, in-house fiber coupler; and DF, source delivery fiber. (b) Signal collection and detection scheme. Abbreviations: VC, viewing computer; DAC, data acquisition card; CF, collection fiber; DB, detection box; PC, photon counter; BP=641/75 nm bandpass filter, SP=750 nm shortpass filter; CL, collimating lens; and CF, collection fiber. (c) Optical probe schematics with insets of contact-based fiber-probe tip (PT) and a tissue sample used in the study (TS). P1 inset corresponds to the contact-based probe without micro-optical components and P2 inset corresponds to the GRIN terminated distal tip focusing probe. Abbreviations: CF, collection fiber; DF, source delivery fiber; PB, probe base; NC, needle chamber; SP, spacer; GL, multimode GRIN fiber lens; and P1 is bare probe without micro-optical components. P2 is the distal-tip focusing probe with micro-optical focusing components.

To combine the two lasers, an in-house fiber coupler was fabricated. For pulse delivery, an FC/APC terminated, 2 m, singlemode fiber was connected directly to the coupler (SM800 or 780HP, Thorlabs). Signal detection was collected and transported to the photon counter detector (H8259-02, Hamamatsu) using an adjacent 2 m multimode fiber (FG105UCA, Thorlabs). For the distal tip focusing probes, a spacer and various lengths of multimode GRIN fibers (GIF625, Thorlabs) spliced to the tip of the singlemode delivery fiber (see Fig. 1 PS). We made many fibers with GRINs of varying focal lengths, and as expected, the closer the focus was to the fiber tip, and therefore the tighter the focal spot, the better the signal generation was. That said, even with focal points as far as 600  μm away from the fiber tip, GRIN-terminated fibers could produce signal comparable with that created with the bare singlemode fiber. The results presented for the GRIN-terminated probe has a focal distance of about 400  μm. All the fibers used had an outer diameter of 125  μm, giving the optical probe an outer diameter of 250  μm and their graphical representation can be seen in Fig. 1. Data were acquired using a homemade system in MATLAB, which decoded the temporally encoded CARS signal from the photon counter (H8259-02, Hamamatsu) using timing outputs from the laser system and plotted the processed spectra in real-time.

Fiber length was an important constraint when designing the CARS probe due to two reasons: (1) shorter pulse-widths and higher peak pulse powers cause faster pulse deterioration through increased FWM in the fiber and (2) the dispersive nature of the fiber requires one to adjust the fiber lengths and delays to obtain overlapped pulses at the sample tip of the fiber and not the connector tip. Indeed, when the pulses overlap at the fiber connector side, the FWM from the fiber is very strong; however, if the pulses overlap at the sample side of the fiber, the FWM from the fiber is minimized. Due to our system’s long pulse widths, we were able to produce CARs spectra even after 10 m of delivery fiber, however, to adequately separate the pulse delay overlap from the fiber connector port and the fiber tip in the sample, at least 2 m of fiber was required. Shown in the Appendix, while the intensity of the FWM background from within the fiber varies greatly depending on which tip of the fiber the pulses overlap, the spectrum of the FWM background signal remains constant. The importance of the background FWM intensity as a function of delay overlap has been mentioned previously in several publications.^9^^,^^12^^,^^20^

To decrease probe size even further and increase signal detection, we attempted to use an in-house fabricated double-clad fiber. Although the increased signal detection was substantial, we also found a considerable increase in background, which turned out to give a worse overall SNR. This was likely due to the internal reflection of the background CARS generation from within the fiber at the fiber tip.

### 2.3 Animals

Experimental procedures were approved by the Comité de Protection des Animaux de l’Université Laval, in accordance with the Canadian Council on Animal Cares Guide to the Care and Use of Experimental Animals. Maximum efforts were made to minimize the number of animals used. As such, we used only two macaque brains from the brain bank we have on hand at the CERVO brain research center. At the time of death of the animals, the brains were fixed by immersion in 4% paraformaldehyde for 24 h at 4°C and later stored in a phosphate buffer solution.

## 3 Results and Discussion

### 3.1 Fiber-Based CARS Spectra from Solutions for System Characterization

**Fig. 2:**
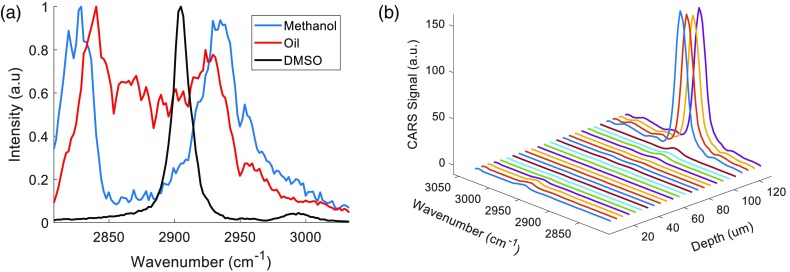
Fiber-based CARS spectra of pure liquids and probe resolution (a) normalized backward-detected spectra using the contact-based probe and (b) resolution characterization using motorized actuator to take spectra every 5  μm, stepping the GRIN-terminated probe toward the DMSO solution.

To show that we were indeed capable of acquiring spectra with minimal background from the fiber, we inserted our two probes into bundle into pure solutions of DMSO, methanol, and peanut oil deposited on cover slips, seen in Fig. 2(a). The data we acquire matched well with previous reports of such solutions using SRS or CARS hyperspectral microscopy, however until now, CARS spectroscopy using a fiber optic delivery has not been presented.^7^^,^^21^ Importantly, in previous work performing CARS endoscopy, it was stated that nonresonant CARS arising from the fiber overpowered the CARS signal generated in a sample of DMSO.^9^ In our experiments, this was not the case. We postulate that this dominant FWM background is due to the previous works’ use of a 280 fs pulsewidth for the pump laser, which is two orders of magnitude shorter than the pulsewidths in our arrangements, and therefore is subjected to a much different level of non-linear effects within the fiber. It is important to note that there are no free space optics between the fiber and the sample, and the signal comes only from the light that is reflected off the glass coverslip on which the solution was stored via Fresnel reflectance. For these experiments, pump and Stokes laser powers were 60 and 20 mW, respectively. The spectral resolution of the wavelength tuning was $2\;{\mathrm{cm}}^{-1}$ and therefore the total acquisition time for all the measurements was 121 ms. As we will show later, this acquisition time can be easily decreased using a lower spectral resolution.

We confirmed our high-resolution spectroscopy by stepping the probes toward DMSO solutions, but this time recording the CARS signal in both the forward direction through the glass coverslip and the backward direction using the probe to assure that our detected resolution was not affected by interface changes. For both probes, the axial and lateral resolution was under 5  μm, the smallest accurate step-size of our stepping motor. The results from the backward detected signal using the GRIN-terminated probe can be seen in Fig. 2(b).

### 3.2 Fiber-Based CARS Spectra of Primate Brain Tissue

**Fig. 3:**
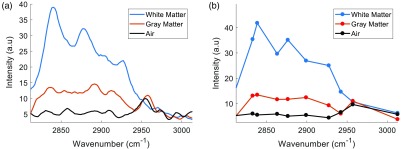
CARS spectra from primate brain sections. The bare probe was manually inserted into white and gray matters and measurements were taken. (a) Spectra acquired in 121 ms with 0.2 nm spectral resolution. (b) Spectra with only 10 random access wavelengths positions. Air measurements are taken with the probe just above the tissue background signal at a realistic intensity.

Pure, non-organic samples are interesting for calibrating a Raman system due to their high signal and known spectra; however, impure scattering media is often much more complicated to achieve good signal from in practice. We show here that the probe could indeed be translated to scattering, multi-molecular tissue, by acquiring spectra in fixed NHP brain tissue as seen in Fig. 3. Again, both probes provided identical results and so we only show here data in this section from the contact-based probe. The power at the tip of the probe was 80 mW total (60 mW at pump wavelength, 20 mW at Stokes wavelength). As tissue gives a much lower signal than pure solutions and we detect only the scattered photons from the forward generated CARS, the background signal from this specific fiber (SM800, Thorlabs) now becomes apparent with a peak signal at $2950\;{\mathrm{cm}}^{-1}$. We can conclude that it is a resonant signal coming from the fiber itself with an irrelevant molecular origin, as it appears when the delay is overlapped at the injection side of the fiber and at the sample side when no sample is present, see the Appendix for more information. Thankfully, this signal is removable and actually can be used to help discern increases in detected signal due to classical scattering from CARS signal increases from denser molecular concentrations. We present two scenarios wherein we acquire high-resolution spectra in a total of 121 ms and a low resolution, random-access version that can be acquired in a total of 10 ms by limiting the number of spectral measurements. This exhibits a major strength of WS-CARS, in that random access contrasting can be achieved at very high speeds if the molecular bonds present are known. To assure the acquired spectra were not affected by the fixation solution used to preserve the tissue [4% paraformaldehyde (PFA)] and the storage solution (0.1 Mol phosphate buffer solution), spectra were taken of each over a mirror and had no contributions in this wavelength range. That said, the application of PFA may cause a slight alteration to the Raman information from the tissue, however, our goal here was just to differentiate tissue subtypes and therefore the reported slight variations caused by PFA are acceptable.^22^ The data presented have been digitally smoothed slightly using a Savistky–Golay filter. As expected, there is a general increase in CARS generated from 2830 to $2950\;{\mathrm{cm}}^{-1}$ in both white and gray matters, with a clear peak at $2845\;{\mathrm{cm}}^{-1}$ appearing only in white matter; this is in accordance with this peak coming from ${\mathrm{CH}}_{2}$ bonds, and those bonds being much more abundant in the myelinated fibers of white matter. We are currently exploring the viability of CARS to provide a fingerprint type spectra for a given brain region and will be presented in future work.

### 3.3 Fiber-Based CARS Spectra Acquired during Descent through Primate Brain

**Fig. 4:**
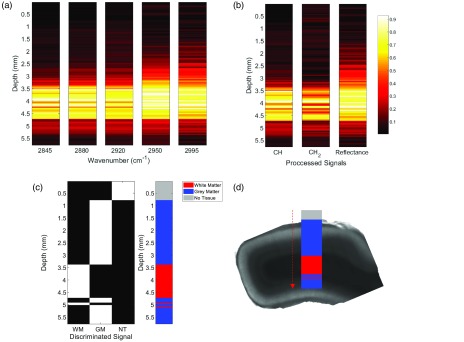
Tissue discrimination using a selection of five wavelengths along a probe trajectory through fixed primate cortex. (a) Heatmap of the intensity from each wavenumber logged when descending from air to gray matter to white matter. (b) Heatmap of processed data showing the relative increase in overall CARS signal deriving from all CH bonds, ${\mathrm{CH}}_{2}$ bonds specifically, and reflectance of fiber background. The maps in (a) and (b) are normalized from 0 to 1 in each graph for display purposes. (c) Thresholded processed data to discriminate different tissue subtypes based on the five wavelengths acquired. WM, white matter; GM, gray matter; and NT, no tissue sensed. (d) Overlay of the detected tissue subtypes on transmission image of primate cortex sample after removal from intact brain.

As a final proof of concept for the future applications of this technology in surgical guidance, we performed a scan through the cortex of an intact primate brain. We show the measurement and discrimination can be done with a selection of only five wavelengths yielding a 5 ms acquisition time per step, showing the capability of CARS to delineate white and gray matters at high resolution, and at very high speeds. The random access capability of the system shortens the acquisition time and also reduces dramatically the amount of power dissipated in the tissue. The wavelengths chosen were strategically placed on $2845\;{\mathrm{cm}}^{-1}$ (corresponding to ${\mathrm{CH}}_{2}$ backbone lipids, abundant in myelinated structures), $2880\;{\mathrm{cm}}^{-1}$ (corresponding to a variation of vibration from lipids), $2916\;{\mathrm{cm}}^{-1}$ (corresponding specifically ${\mathrm{CH}}_{3}$ found more abundantly in cells due to proteins), $2950\;{\mathrm{cm}}^{-1}$ (corresponding to the resonant background coming from the fiber), and lastly $2995\;{\mathrm{cm}}^{-1}$ acting as the nonresonant contribution. It is important to mention that we expect the signals at 2950 and $2995\;{\mathrm{cm}}^{-1}$ to increase as they do along the probe descent, due to classical scattering as we approach the highly scattering white matter layer. Using this knowledge, we can use these signals as our subtractable background to improve the SNR and tissue classification. For help understanding the chosen wavelengths, refer to Fig. 3. Using these wavelength intensities when passing through different tissues, it becomes trivial to segment the different tissue types, as seen in Fig. 4. Specifically, in Fig. 4(b), the heat maps labeled ${\mathrm{CH}}_{2}$ and ${\mathrm{CH}}_{3}$ are created through the subtraction of the $2995\;{\mathrm{cm}}^{-1}$ signal from the $2845\;{\mathrm{cm}}^{-1}$ and the sum of the 2880 and $2920\;{\mathrm{cm}}^{-1}$ signals, respectively. The reflectance signal is merely the $2995\;{\mathrm{cm}}^{-1}$ itself as it is wavelength independent and is always present regardless of the medium. This reflectance signal is a useful parameter to have not only for baseline correction but also for alluding to distant upcoming structures that can then be confirmed by a resonant CARS signal, such as the ${\mathrm{CH}}_{2}$ signal as a white matter fiber bundle approaches in this example. Reflectance measurements like this have been used in the past for DBS optical guidance using large probes that do not fit within the DBS electrode.^23^ The white matter and gray matter segmentation performed in Fig. 4(c) is done using a high threshold for $2845\;{\mathrm{cm}}^{-1}$ and a low threshold for the sum of 2880 and $2920\;{\mathrm{cm}}^{-1}$. To avoid co-registered tissues, we also restrict that if white matter is sensed, the structure is not gray matter. The “no tissue” segmentation occurs when the sum of all the lipid-originating signals is near zero. Although we performed these tests with both probes, the data presented here are with the GRIN-terminated probe. The probe was placed within a 26 gauge needle for rigidity, with the tip of the fiber slightly offset within tip of the needle to have the focal volume just outside the metal tube.

## 4 Discussion

Although the system is a considerable technical advancement in the field of fibered CARS spectroscopy, there are many future improvements we hope to incorporate to truly fulfill its potential. The first will be to perform the spectroscopy from within a DBS electrode. This will prove to be a difficult step, due to the tip of the DBS electrode not being perfectly transparent and therefore our signal detection will suffer both from the loss of ballistic photons and reduced collection due to interface reflections. That said, we have started to look at all the various commercially available electrodes that are FDA approved to give the system the optimal SNR. Another endeavor we are currently working on is acquiring fingerprint-type spectra from specific brain regions, as has previously been done with spontaneous Raman probes, but with a much higher resolution.^19^ Furthermore, CARS offers the potential to perform this fingerprint-type sensing on the other side of the electrode with, theoretically, one or two orders of magnitude faster speeds depending on the spectral resolution required, comparing with spontaneous Raman probes of a similar size.^19^ This decrease in speed may not be so important in the operating room; however, the resulting decrease in optical power deposition in the tissue is highly desirable.

While white and gray matter can be a powerful enough tool to guide the surgery and provide locational information of the electrode within the brain,^23^ a fingerprint spectra would provide much more valuable information. Currently, in DBS surgery, a microelectrode recording (MER) is routinely done before electrode implantation wherein a microelectrode is descended into the brain, and then removed before implanting the chronic electrode. This is performed as MER can give a form of fingerprint-type sensing of the STN due to its unique neuronal activity pattern; therefore, this can confirm for the surgeon their planned implantation trajectory is on target. If we can perform the same fingerprint sensing at an even higher resolution, optically, all from within the chronic electrode, we could dramatically improve the surgical outcome not only by guiding the placement of the electrode, but also by removing the need for multiple foreign objects sent deep within the brain.

## 5 Conclusion

We presented here the first all fiber-based CARS spectroscopy system using simple silica fiber optics and a fast wavelength-tuning fiber-laser. With this system we have designed and created a probe with an ultra-small form factor, capable of deep brain tissue sensing. We show that our laser system has the capability to be portable while also having clinically relevant fiber lengths. As a relevant proof of concept, we show, to the best of our knowledge, the first fiber-based CARS sensing of primate brain tissue. Furthermore, we show the ability of this spectroscopic technique to tell white and gray matter boundaries with very high-resolution and acquisition speeds, alluding to its usefulness in DBS optical guidance.

## Figures and Tables

**Fig. 5:**
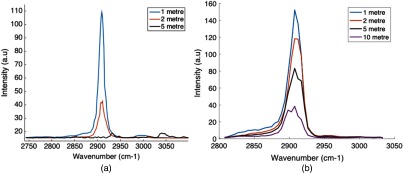
CARS spectrum as a function of fiber length (a) OPO laser used experiments stopped at 5 m as no signal was visible after this length and (b) Genia laser system used. DMSO spectra still visible after 10-m fiber length.

**Fig. 6:**
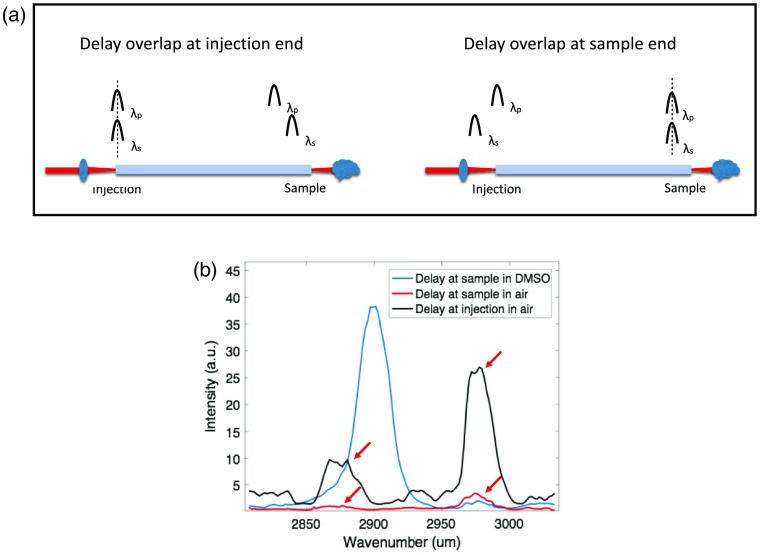
CARS background from fiber over wavelength range. Spectra taken with detector directly in front of fiber, or through thin layer of DMSO in the case of DMSO (a) schematic to understand what is meant by pulse delay overlap. In the dispersive silica media, the two wavelengths travel at different speeds, allowing us to separate the pulses at one end of the fiber. (b) Example of CARS spectra with different delay overlap positions in both air and in DMSO for comparison.

## Appendix

### A.1 CARS Spectra as a Function of Fiber Length and Laser System

High-power peak pulses required for CARS signal generation deteriorate as a function of the pulse width. To show the improvement our system had over shorter pulse lasers, we aligned our labs CARS microscope lasers into the fiber probe. This laser system is composed of an optical parametric oscillator (OPO) (APE, Levante Emerald) pumped by a frequency-doubled Nd:YVO4 mode-locked laser (High Q Laser, picoTRAIN). The pump laser generates a 7 ps, 80-MHz pulse train of 532- and 1064-nm laser light. The OPO utilizes a temperature-tuned LBO nonlinear crystal for parametric oscillation and is pumped with 5.5 W of 532-nm laser light. In our application, we used a fraction of the 1064-nm laser light in combination with a small portion the tunable wavelength range of the OPO (803 to 820 nm) to produce CARS spectra in the high wavenumber region (2800 to $3050\;{\mathrm{cm}}^{-1}$).

Indeed, using 7-ps pulses from the OPO laser system, we found a rapid degradation of the DMSO CARS spectra with fibers >1 meter long; however, with the 25-ps pulses output from the Genia laser system, we could observe DMSO spectra with fibers’ lengths up to 10 m, as seen in Fig. 5. This is important when considering clinical translatability, as the possibility of longer fiber lengths is ideal.

### A.2 Background CARS Generation from Delivery Fiber

We found that the FWM in the fiber was small in comparison with the signal generated in the sample when the delay between the two lasers was zero at the sample end of the fiber. In Fig. 6(b), we show that when the delay between the two different lasers is zero at the sample side of the fiber the background is small compared with the DMSO; however, if the delay is zero at the injection side, the intensity of the fibers’ FWM background is high. When the pulses are overlapped at the injection side and the sample-side tip is in DMSO, no DMSO is detected as expected. In our work, we were able to reproducibly get the same wavelength-dependent background spectra from a given fiber, in this example a singlemode, polarization maintaining from Thorlabs (780HP, Thorlabs) was used.
